# Do Skin Symptoms of Viral Infections Tend to Worsen and Spread to the Limbs? The Role of Venous Pressure in Rash Exacerbation

**DOI:** 10.1002/ccr3.70804

**Published:** 2025-09-24

**Authors:** Yukako Nagano, Seimi Watanabe, Keiichi Yamanaka

**Affiliations:** ^1^ Department of Dermatology, Graduate School of Medicine Mie University Tsu Mie Japan

**Keywords:** Epstein–Barr virus infection, limb, skin rash, venous pressure

## Abstract

Viral infections may cause disseminated erythema that typically originates on the trunk but often shows peripheral extension and exacerbation; increased venous pressure dynamics may play a role in the exacerbation of viral eruption in the limbs.

One clinical manifestation of viral infections is a skin rash. While rashes typically originate on the trunk, they extend and worsen on the limbs. However, the underlying reason for this phenomenon remains unclear.

An 18‐year‐old female with primary Epstein–Barr virus infection developed disseminated erythema. One day, blood sampling was performed from the left cubital fossa via the ulnar median basilic vein, during which the tourniquet time on the left upper arm was prolonged. The following day, the eruption on the left upper limb showed marked exacerbation compared to the right upper limb (Figure 1A). The rash on the lower limbs remained similarly severe (Figure 1B). The venous pressure in the limbs often exceeds that of the trunk [1, 2]. Further investigation is required to validate, but we hypothesize that peripheral extension and exacerbation of the virus cutaneous eruption may be in response to increased venous pressure dynamics in the limbs.

**FIGURE 1:**
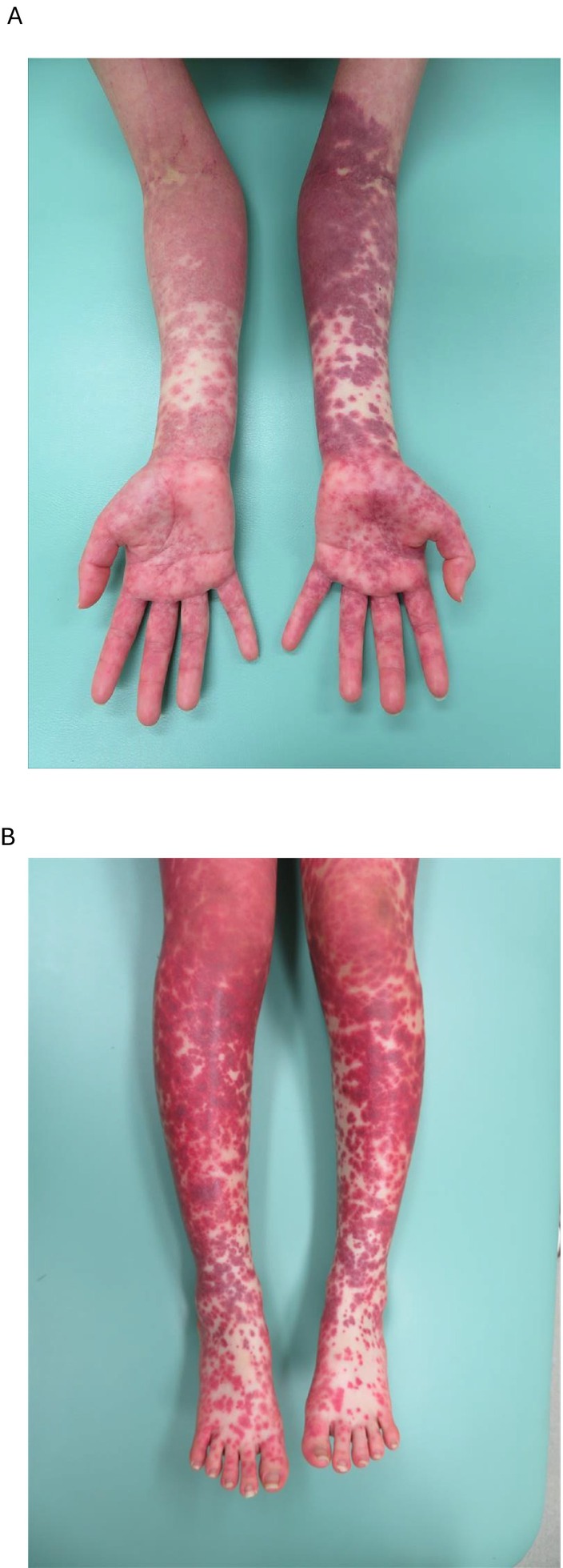
(A) The disseminated erythema caused by primary Epstein–Barr virus infection was shown. On one occasion, blood sampling was performed from the left cubital fossa via the ulnar median basilic vein, during which the tourniquet time on the left upper arm was prolonged. The following day, the rash on the left upper limb showed marked exacerbation compared to the right upper limb. (B) The rash on the lower limbs remained similarly severe.

## Author Contributions

**Yukako Nagano:** conceptualization, writing – original draft. **Seimi Watanabe:** conceptualization, writing – original draft. **Keiichi Yamanaka:** conceptualization, writing – original draft, writing – review and editing.

## Ethics Statement

The study was conducted in accordance with the Declaration of Helsinki. The patient provided written informed consent to publish the case, including the publication of images. The paper is exempt from ethics committee approval as only one case was reported.

## Consent

Written consent for publication was obtained from the patient.

## Conflicts of Interest

The authors declare no conflicts of interest.

## Data Availability

The patients data is not publicly available on legal or ethical grounds.

## References

[1] K. Giakoumidakis , A. Patelarou , A. A. Chatziefstratiou , M. Zografakis‐Sfakianakis , N. V. Fotos , and E. Patelarou , “Development and Validation of the CVP Score: A Cross‐Sectional Study in Greece,” Healthcare (Basel) 11 (2023): 1543. 37297683 10.3390/healthcare11111543 PMC10252753

[2] S. Raju , A. Knight , L. Lamanilao , N. Pace , and T. Jones , “Peripheral Venous Hypertension in Chronic Venous Disease,” Journal of Vascular Surgery. Venous and Lymphatic Disorders 7 (2019): 706–714. 31196767 10.1016/j.jvsv.2019.03.006

